# Fluorescence Resonant Energy Transfer-Based Quantum Dot Sensor for the Detection of Calcium Ions

**DOI:** 10.3389/fchem.2020.00594

**Published:** 2020-08-11

**Authors:** Shreya Ghosh, Yinghua Chen, Anne George, Mitra Dutta, Michael A. Stroscio

**Affiliations:** ^1^Micro and Nanotechnology Laboratory, University of Illinois at Urbana-Champaign, Urbana, IL, United States; ^2^Department of Oral Biology, University of Illinois at Chicago, Chicago, IL, United States; ^3^Department of Electrical and Computer Engineering, University of Illinois at Chicago, Chicago, IL, United States; ^4^Department of Physics, University of Illinois at Chicago, Chicago, IL, United States; ^5^Department of Bioengineering, University of Illinois at Chicago, Chicago, IL, United States

**Keywords:** aptamer, biosensor, calcium detection, FRET, quantum dot, optical sensor

## Abstract

A simple optical aptasensor has been synthesized for the detection of calcium ions. This sensing approach employs a semiconductor quantum dot (QD)–gold nanoparticle as the donor–quencher pair and operates on the principle of fluorescence resonant energy transfer (FRET). On binding with calcium ions, the DNA aptamer undergoes a conformational change, which changes the distance between the quantum dot and the gold nanoparticle, conjugated on the 5′ terminal and 3′ terminal of the aptamer, respectively. This phenomenon results in the quenching of the quantum dot emission. In this sensor, a maximum quenching of 22.42 ± 0.71% has been achieved at 35 nM calcium ion concentration while the limit of detection has been determined to be 3.77 pM. The sensor has been found to have high specificity for calcium ions in comparison to other metal ions like sodium, magnesium, and potassium. The molecular apta-beacons also demonstrated successful endocytosis and FRET-based calcium ion detection in osteocyte cells when conjugated with a cell-penetrating peptide (DSS).

## Introduction

**Figure 1:**
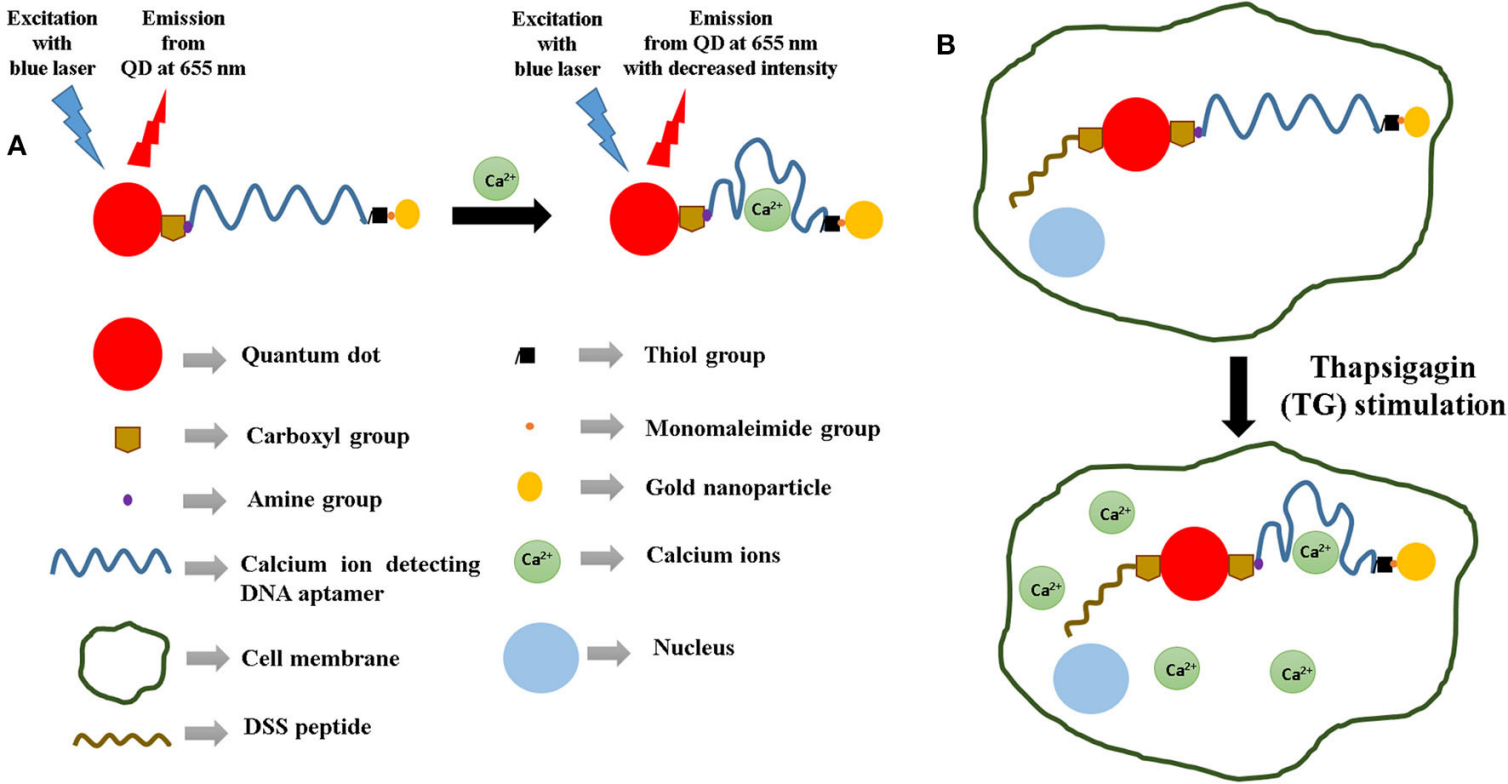
**(A)** Sensing strategy used for the detection of calcium ions using DNA aptamers and the quantum dot–gold nanoparticle FRET pair. **(B)** Illustration of the strategy used for the aptasensor-based detection of calcium ions inside cells. In this case, a cell-penetrating peptide (DSS) is used to endocytose the sensors within the cells.

Calcium ions (Ca^2+^) are an essential component of the physiological system. They play a significant role as an intracellular messenger, which regulates several cellular functions like secretion, contraction, excitability, and gene expression (Russell, 2011). An increased Ca^2+^ release can contribute to diseases like HIV, schizophrenia, and Alzheimer's disease (Wojda et al., 2008). The review by Feske et al. throws light on the role of Ca^2+^ signaling in congenital immunodeficiency syndromes along with autoimmunity and inflammatory conditions (Feske, 2007). For instance, in systemic lupus erythematosus (SLE), it has been observed that signaling through the B-cell receptor in B cells is abnormal and results in increased Ca^2+^ signals. Chung et al. reported that with an elevation in the Ca^2+^ concentration, there was a greater risk of long-term mortality after an acute ischemic stroke (Chung et al., 2015). Hence, owing to the significance of this metal ion in the physiological system, the objective of this study is to design a sensor, which rapidly detects Ca^2+^. Asif et al. reported a zinc oxide nanorod-extended gate field-effect transistor (MOSFET), which detected Ca^2+^ linearly between 1 μM and 1 mM (Asif et al., 2009). Several analytical techniques for Ca^2+^ sensing have been published in literature. Ankireddy designed an optical sensor consisting of highly fluorescent ethylenediaminetetraacetic acid (EDTA)-CDs (ECDs) to detect Ca^2+^ in human serum with a detection limit of 77 pM (Ankireddy and Kim, 2018). Calsequestrin-functionalized gold nanoparticles were employed by Kim et al. to detect Ca^2+^ colorimetrically in human serum (Kim et al., 2009). Asadnia et al. used an AlGaN/GaN transistor functionalized with poly(vinylchloride) (PVC)-based membranes as a sensing platform for Ca^2+^ (Asadnia et al., 2017). Other methods include potentiometric detection (Ganjali et al., 2005; Singh and Mehtab, 2007), microfluidic chips (Caglar et al., 2006), and ion-selective electrodes (Schefer et al., 1986). Although in the last few years the development of aptamer-based sensors for various kinds of target detection has attracted huge interest, they have been hardly explored in the field of Ca^2+^ detection. Aptasensors utilize short single-stranded DNA/RNA oligonucleotides to bind to a specific target molecule. The binding affinity can be transduced using various methods and therefore can be employed as a primary sensing component in several types of sensors. In this manuscript, we have designed a DNA aptamer-based optical sensor for the detection of calcium ions. This sensor is based on the principle of FRET, which changes the photoluminescence (PL) intensity of the semiconductor quantum dot crystal depending on its distance with the gold nanoparticle quencher (Figure 1A).

The phenomenon of fluorescence resonance energy transfer (FRET) facilitates transfer of energy from a “donor” to an “acceptor.” In the process of FRET, the efficiency is proportional to 1/{1 + (d/d_o_)^6^}, where d is the distance between the donor and the acceptor and d_o_ has been determined to be approximately 5 nm (Markvart, 2000; Yun et al., 2005; Shu et al., 2013). This distance-dependent behavior occurs due to dipole–dipole interactions between the donor and the acceptor. The effect of FRET is relatively strong when d is less than about 5 nm and starts getting weaker when d is greater than about 5 nm. This study incorporates a quantum dot as the donor and a gold nanoparticle as the acceptor. Variation of distance between the QD and the nanoparticle quencher causes a transfer of energy from the QD to the nanoparticle. When d between QD and the nanoparticle acceptor is less than 5 nm, there is less energy available in the QD to emit as photons because of a strong transfer of energy from it to the quencher. Consequently, the QD light emission decreases significantly. The sensor design involves the QD donor and the gold nanoparticle acceptor conjugated to opposite ends of a DNA aptamer, which functions as the primary sensing element here. This is because the aptamer has the ability to change its conformation on binding to the target analyte. As a result of the change in the conformational shape of the aptamer, there is a variation in d, which further causes a change in the intensity of the light emitted by the QD. Therefore, this change in QD emission can be considered as an indicator of d as well as the binding between the aptamer and the target. This phenomenon has been used as the principle of Ca^2+^ detection in this molecular beacon-based sensing platform.

In the recent years, quantum dots of various types of composition (Qi et al., 2016; Jia et al., 2020) have attracted huge attention because of their applicability in a wide variety of biomedical applications (Wegner et al., 2019; Song et al., 2020). We have successfully detected biomarker proteins like glycated albumin (Ghosh et al., 2017) and tumor necrosis factor-alpha (Ghosh et al., 2018) using this design strategy. Apart from biomarker proteins, our group has also used this sensing strategy to detect metal ions like potassium (Wu et al., 2011; Meshik et al., 2014), lead (Brenneman et al., 2013; Meshik et al., 2014), and mercury (Brenneman et al., 2010). The innovation of this study lies in the design of the sensor, which has a cell-penetrating peptide conjugated to the aptamer-based FRET sensor. Such a sensor for the detection of calcium ions has not been reported in literature previously. Therefore, this paper also investigates the response of a cell-penetrating peptide (DSS)-conjugated aptasensor inside a cellular environment and its effectiveness in detecting intracellular Ca^2+^ (Figure 1B). Such aptasensors have the potential of being incorporated in point-of-care setups for clinical applications.

## Materials and Methods

### Materials Used for Synthesis and Testing of Molecular Beacon

The calcium-detecting DNA aptamer was purchased from Biosearch Technologies (Petaluma, CA). Calcium chloride dihydrate (CaCl_2_·2H_2_O), 2 M magnesium chloride (MgCl_2_) solution, and 5 M sodium chloride (NaCl) solution were purchased from Sigma-Aldrich (St. Louis, MO) while 4.6 M potassium chloride (KCl) solution was obtained from Fischer Chemicals (Fairlawn, NJ). 1-Ethyl-3-(3-dimethylaminopropyl)-carbodiimide (EDC) and tris(2-carboxyethyl)phosphine (TCEP) were purchased from Pierce Biotechnology (Rockford, IL). Monomaleimide-functionalized nanogold particles (diameter = 1.4 nm) were obtained from Nanoprobes (Yaphank, NY). Carboxyl-coated CdSe/ZnS QDs e-flour ITK 655NC (diameter = 20 nm) was obtained from Life Technologies (Carlsbad, CA). Nanosep molecular weight cutoff (MWCO) filters of 3 and 100 k pore sizes were purchased from Pall Life Sciences (Ann Arbor, MI).

### Aptamer Structure and Preparation of Aptamer Stock Solution

The calcium-detecting DNA aptamer consisted of 12 bases and had been modified with an amine group on the 5′ terminal and a thiol functional group on the 3′ terminal (5′-amino C6/GGGGTTTTGGGG/thiol C6 SS 3′). The aptamer was dissolved into 654 μl of tris ethylenediamine tetraethyl acetate (EDTA) buffer to obtain 100 μM aptamer stock solution in order to prevent cation-induced degradation.

### Preparation of Molecular Beacon

The molecular beacon was synthesized based on the protocol reported by Ghosh et al. (2017, 2018). Briefly, 9 μl of TCEP was added to 20 μl of the 100 μM calcium-detecting aptamer. The mixture was allowed to incubate for 30 min at room temperature so that the dithiol groups in the aptamer get reduced. One vial of gold nanoparticles (6 nmol) was added to 100 ml of deionized water to form a solution, which was further added to the aptamer–TCEP mixture (quencher: aptamer = 3:1 approximately). This mixture was then incubated for 2 h at room temperature, after which it was centrifuged [Fisher Scientific accuSpin Micro (Fisher Scientific, USA)] twice at 5,000 rpm for 15 min each using a 3 k MWCO filter. This step ensured the removal of excess unbound gold nanoparticles from the mixture. The supernatant after each centrifugation was washed with 50 μl of deionized water. 13 μl of carboxylated CdSe/ZnS QDs (0.1 nmol) was mixed with 87 μl of 10 mM borate buffer (pH 7.4) to form a 100 μl QD solution, which was further added to the filtered DNA aptamer/gold nanoparticle solution in the presence of 23 μl of 4 μg/μl EDC/Sulfo-NHS solution. The resulting mixture was then allowed to shake gently for 2 h at room temperature, following which the samples were centrifuged five times at 7,000 rpm for 5 min each using a 100 k MWCO filter in 50 mM borate buffer (pH 8.3). The supernatant left after each centrifugation was washed with 50 μl of the 50 mM borate buffer (pH 8.3). This resulted in the removal of unbound aptamers and EDC from the sensor solution.

### Preparation of DSS Peptide-Conjugated Molecular Beacons for Calcium ion Sensing

9 μl of TCEP was added to 20 μl of the 100 μM calcium-detecting aptamer. The mixture was allowed to incubate for 30 min at room temperature so that the dithiol groups in the aptamer get reduced. One vial of gold nanoparticles (6 nmol) was added to 100 ml of deionized water to form a solution, which was further added to the aptamer–TCEP mixture (quencher: aptamer = 3:1 approximately). This mixture was then incubated for 2 h at room temperature, after which it was centrifuged (Fisher Scientific accuSpin Micro [Fisher Scientific, USA)] twice at 5,000 rpm for 15 min each using a 3 k MWCO filter. The supernatant after each centrifugation was washed with 50 μl of deionized water. 13 μl of carboxylated CdSe/ZnS QDs (0.1 nmol) was mixed with 87 μl of 10 mM borate buffer (pH 7.4) to form a 100 μl QD solution. A 10-mg/ml DSS peptide solution was prepared by adding 2.3 mg of the DSS peptide to 230 μl of deionized water. 30 μl of 4 μg/μl EDC/Sulfo-NHS solution was added to a mixture of 100 μl of the QD solution, 230 μl of the DSS peptide, and the filtered calcium aptamer/gold nanoparticle solution in order to facilitate binding. Subsequently, this mixture was allowed to shake for 2 h at room temperature, following which the samples were centrifuged five times at 7,000 rpm for 5 min each using a 100 k MWCO filter in 50 mM borate buffer (pH 8.3). The supernatant obtained after each centrifugation cycle was washed with 50 μl of the 50 mM borate buffer (pH 8.3).

### DNA Secondary Structure Determination

The secondary structure of the calcium ion-detecting DNA aptamer was predicted using the M-fold web server (SantaLucia, 1998; Peyret, 2000; Zuker, 2003). The predicted secondary structures were compared at different temperatures and sodium ion (Na^+^) concentrations. The temperatures used are as follows: (1) 20°C: This has been considered as the room temperature and the temperature in which the optical characterization experiments were conducted (2) 37°C: This is the temperature under physiological conditions. There were three different ionic conditions considered: (1) 1.37 mM Na^+^, (2) 10 mM Na^+^, and (3) 150 mM Na^+^. These sodium ion concentrations were chosen because the Na^+^ concentration for this aptasensor is approximately 1.37 mM while the concentration of Na^+^ in a mammalian cell and blood is around 12 and 145 mM, respectively (Lodish et al., 2000).

### Sensitivity Determination of Sensor

The 1 M CaCl_2_ stock solution was prepared by adding 1 g CaCl_2_ to 10 ml deionized water. This stock solution was serially diluted to obtain solutions having concentrations of 0.7 nM, 3.5 nM, 7 nM, 35 nM, 0.7 μM, and 3.5 μM, respectively. These solutions were used as working solutions. 5 μl of these working solutions was added to 750 μl of the sensor solution in the cuvette, which was then allowed to stand undisturbed for 5 min. This time period ensured the binding of the calcium ion to the DNA aptamer in the sensor. The photoluminescence intensities were subsequently recorded using a USB4000 Ocean Optics (Dunedin, FL, USA) spectrophotometer with a continuous 375-nm LED excitation.

### Specificity Determination of Sensor

One-mM stock solutions of the control analytes (NaCl, MgCl_2_, and KCl) were obtained by serially diluting the respective 5 M NaCl, 2 M MgCl_2_, and 4.6 M KCl solutions. The respective stock solutions were then serially diluted to obtain 1 μM and 100 μM working solutions. These working solutions were added to the sensor solution in such a way that the final concentration of the control analytes were 660 pM, 7 nM, 600 nM, and 7 μM. The PL spectra corresponding to the controls were recorded after 5 μl of the control ion was added to 750 μl of the sensor solution and allowed to stand disturbed for 5 min.

### FRET Determination in an Intracellular Environment

Mouse pre-osteocyte cells (MC3T3 E1—ATCC, Manassas, VA) were cultured in α-MEM (Corning Inc., Corning, NY) with 10% FBS (Thermo Fisher Scientific, Waltham, MA) and 1% antibiotic–antimycotic (100×, Life Technologies) at 37°C in a humidified incubator with 5% CO_2_. The 300,000 cells were seeded on a φ 25-mm cover glass in a well of a 6-well culture plate. The next day, the DSS-conjugated molecular beacons (0.1 mg/ml) were added. After 1 h, the cells were washed with pre-warmed PBS without calcium and magnesium three times to remove un-incorporated/free molecular beacons. Then, thapsigargin (TG—final concentration at 1 μM) [MilliporeSigma, Burlington, MA)] in PBS without calcium and magnesium was added to trigger the calcium release from endoplasmic reticulum storage. At the indicated time point, formaldehyde solution (37%) (Thermo Fisher Scientific, Waltham, MA) was added at 1/10 of TG solution volume (20 μL to 200 μL) to stop the reaction and fix cells for 1 h at room temperature. After washing with PBS for 3 times, the cover glass was mounted on a glass slide with a mounting agent with DAPI (Vector Lab, Burlingame, CA). The fluorescence signals from the molecular beacons were observed with a Zeiss LSM 710 Confocal Microscope in Research Resources Center of University of Illinois at Chicago.

## Results and Discussion

### Aptamer Structure

**Figure 2:**
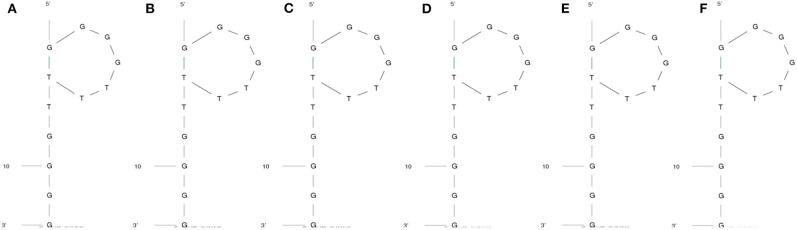
Secondary structure of the aptamer when the temperature and Na^+^ concentrations are **(A)** 20°C and 1.37 mM, **(B)** 37°C and 1.37 mM, **(C)** 20°C and 10 mM, **(D)** 37°C and 10 mM, **(E)** 20°C and 150 mM, and **(F)** 37°C and 150 mM, respectively.

The secondary structure of the DNA aptamer was found to be similar under all conditions mentioned in Figures 2A–F. As predicted by the M-fold web server, the secondary structure had a characteristic external loop and a hairpin loop. The external loop was composed of 5 single-strand bases along with 1 closing helix. On the other hand, the hairpin loop had a closing pair at G^1^-T^7^. As shown in Table 1, Gibb's free energy was observed to increase with an increase in temperature while no such pattern was observed for the sodium ion concentration. The aptamer was chosen from the work reported by Miyoshi et al. (2003). They determined that the DNA aptamer underwent a structural transition from antiparallel to parallel G-quadruplex in the presence of Ca^2+^. This characteristic was utilized to induce FRET in the proposed sensor here.

**Table 1:** Gibbs-free energy values of the Ca^2+^-detecting DNA aptamer under various Na^+^ concentrations and temperature conditions.

**Serial number**	**Parameters**		**ΔG (kcal/mol)**
	**Temperature (^**°**^C)**	**Na^**+**^ concentration (mM)**	
1.	20	1.37	2.71
2.	37	1.37	2.85
3.	20	10	2.71
4.	37	10	2.85
5.	20	150	2.61
6.	37	150	2.76

### Sensitivity Determination of Sensor

**Figure 3:**
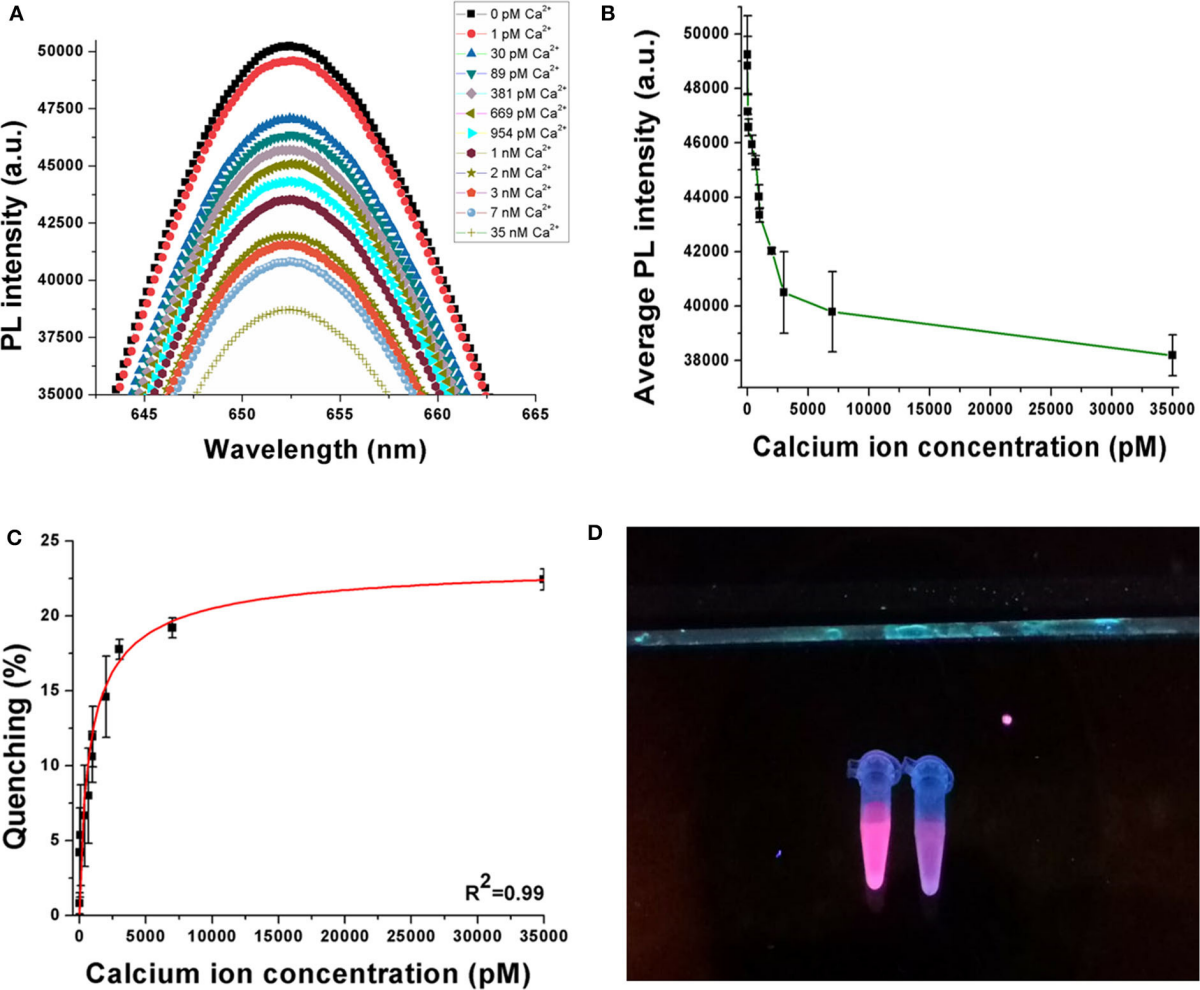
**(A)** Decrease in PL intensity with increase in Ca^2+^ concentration. **(B)** Average PL intensity curve of the sensor when experiments are repeated with multiple samples. **(C)** Quenching behavior of the sensor when the concentration of Ca^2+^ is increased up to 35 nM. **(D)** Visual illustration of quenching phenomena in the sensor on addition of target ion. Experiments have been conducted in quintuplicates (*n* = 5).

A decrease in photoluminescence intensity was observed with an increase in the concentration of the calcium ions. This phenomenon is shown in Figure 3A, where the PL spectra indicate a decrease when Ca^2+^ is progressively added between 0 pM and 35 nM. A consistent repetition of this behavior is observed in Figure 3B when the experiments are repeated in quintuplicates (*n* = 5), where the average PL intensity decreases with an increase in the target ion concentration. Figure 3C shows the quenching behavior of the sensor samples. Quenching (%) has been calculated using Equation (1), where I_blank_ is the peak photoluminescence intensity before the addition of target, I_Ca_ is the peak photoluminescence intensity after the addition of Ca^2+^, and quenching (%) is the quenching efficiency of the sensor. The quenching efficiency is an indicator of the occurrence of FRET in the sensor while detecting the target analyte (Held, 2005).

(1) $$\begin{array}{l} Quenching\;\left(\%\right)=\frac{\left(I_{blank}-I_{Ca}\right)}{I_{Ca}}\times 100 \end{array}$$

According to Figure 3C, the sensor achieves average quenching efficiencies of 4.2 ± 2.97% and 22.42 ± 0.71% at 30 pM and 35 nM Ca^2+^ concentrations, respectively. This behavior can be attributed to the phenomenon of FRET, where the DNA aptamer binds to the target analyte and changes its conformation, because of which the donor (QD) and the quencher (gold nanoparticles) are driven closer to each other. Consequently, owing to a dipole–dipole interaction between the FRET pair, there is an intersystem transfer of energy from the donor to the quencher. This reduces the resulting emission from the QDs, causing a decrease in PL intensity. As the concentration of the target ion is increased, a greater number of DNA aptamers bind to them and hence a higher number of QDs participate in FRET. In this case, the DNA aptamer has been reported to have an antiparallel G-quadruplex structure initially. Addition of Ca^2+^ induces the formation of a parallel G-quadruplex structure and finally to a G-wire structure. The parallel G-quadruplex has been found to be unstable, and hence, the aptamer rapidly transitions to the G-wire structure. The visual illustration shown in Figure 3D shows reduced fluorescence emission from the sample on the right compared to that on the left because the latter has no target analyte added to it while the former has 100 nM Ca^2+^ added. This further establishes the successful occurrence of FRET in the sensor on the addition of the target ion.

According to Figure 3C, the quenching behavior of the nanosensor follows the Hill chemical kinetics. In the Hill formalism, the quenching efficiency can be expressed as:

(2) $$\begin{array}{l} Quenching=23.765\times \frac{x^{0.765}}{\left(906.703^{0.765}+x^{0.765}\right)} \end{array}$$

Based on the definition of Hill's equation, 906.703 pM refers to the concentration at which half of the receptors are occupied by the target. The Hill coefficient of 0.765 is the slope of the Hill curve and also refers to negative cooperativity with respect to substrate binding. Also, from the equation theory, the average binding constant (K_D_) can be estimated as 183 pM.

(3) $$\begin{array}{l} LOD=\frac{3\times SD_{0}}{Sensitivity_{Ca}} \end{array}$$

From the results obtained in Equation (3), where the SD_0_ is the normalized standard deviation of the blank sensor sample while Sensitivity_Ca_ is the slope of the quenching curve (Figure 3C), the limit of detection (LOD) was calculated to be 3.77 pM or 0.55 pg/ml (assuming molecular weight of the CaCl_2_·2H_2_O = 147 g/mol). The LOD obtained from this sensor has been compared with several other sensing platforms for Ca^2+^ in Table 2.

**Table 2:** Comparative summary of various sensing platforms reported in literature for calcium ion detection.

**Sensing element**	**Sensor type**	**Limit of detection**	**References**
Calmodulin (CaM)	Fiber-optic sensor	5 × 10^−8^ M	Blair et al., 1994
Carboxylic polyether antibiotic A23187	Fiber-optic sensor	1 × 10^−7^ M	Suzuki et al., 1989
Ionophore N,N,N′,N′-tetracyclohexyl-3-oxapentanediamide	Calcium-selective electrode	100 pM	Schefer et al., 1986
Sensing membrane consisting of modified merocyanine photoacid polymer and a calcium ionophore in plasticized poly(vinyl chloride)	Optical sensor	5 × 10^−4^ M	Johns et al., 2014
NiCo_2_O_4_ nanostructures on 3-dimensional graphene foam	Electrochemical sensor	4.45 μM	Wu et al., 2015
α-Furildioxime ionophore	Potentiometric sensor	1.25 × 10^−7^ M	Singh and Mehtab, 2007
Arsenazo III (1,8-dihydroxynaphthalene-3,6-disulfonic acid-2,7-bis[(azo-2)-phenyl arsenic acid])	Microfluidic fiber-optic sensor	2.68 × 10^−5^ M	Caglar et al., 2006
2-[(2-Hydroxyphenyl)imino]-1,2-diphenylethanone (HD)	Potentiometric sensor	8.0 × 10^−7^ M	Ganjali et al., 2005
Ethylenediaminetetraacetic acid (EDTA)-carbon dots	Optical sensor	77 pM	Ankireddy and Kim, 2018
Hexametaphosphate-capped CdS QDs	Optical sensor	4 μM	Liu et al., 2016
4,4′,4′′,4′′′-((3,6-Dicyanobenzene-1,2,4,5-tetrayl)tetrakis(sulfanediyl))tetra-benzoic acid	Optical sensor	0.6 μM	Chen et al., 2019
DNA aptamer	Optical sensor	3.77 pM	This work

### Specificity Determination of Sensor

**Figure 4:**
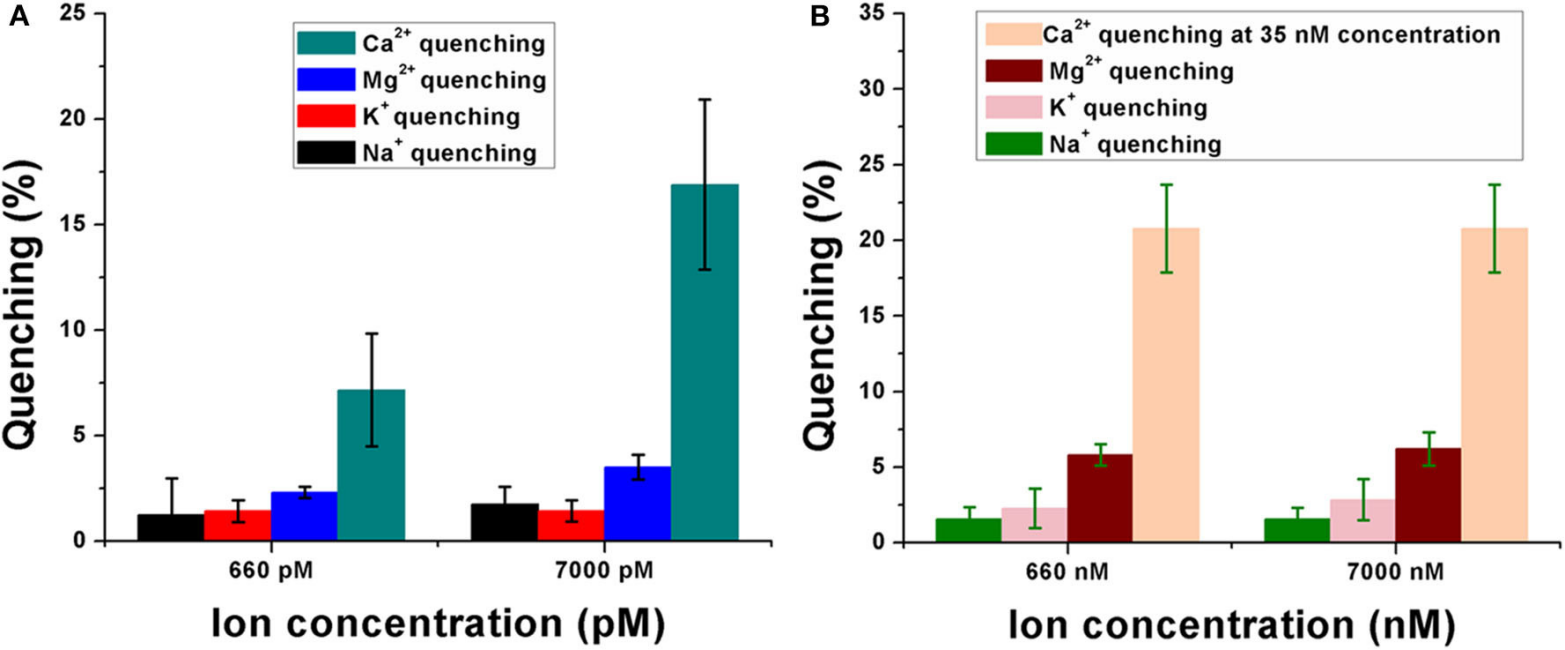
Response of the calcium ion-detecting nanosensor when tested with control ions like sodium (Na^+^), potassium (K^+^), and magnesium (Mg^2+^). Quenching efficiencies of the target ion have been compared with that of the control ions in the pM concentration range **(A)** as well as in the nM range **(B)**. Experiments have been conducted in quintuplicates (*n* = 5).

The sensor was observed to have significant selectivity toward Ca^2+^ when compared to the control metal ions like Na^+^, Mg^2+^, and K^+^. These cations were specifically chosen because of their importance and abundance in the physiological system. Figure 4 shows the quenching behavior of the control ions in the picomolar (pM) and nanomolar (nM) concentration range. In Figure 4A, the quenching efficiencies have been compared between all the four ions while keeping their concentrations the same (660 pM and 7 nM). Ca^2+^ was shown to have a significantly high quenching efficiency compared to Na^+^, Mg^2+^, and K^+^ at both 660 pM and 7 nM concentrations, respectively (Figure 4B), indicating a high selectivity of the sensor toward calcium in the pM concentration range. A similar response was observed even when the concentration of the control ions is much higher (660 nM and 7 μM) in the sensor solution. Their quenching efficiencies were significantly lower than that of Ca^2+^ at 35 nM concentration in the sensor. This further established the specificity of the aptasensor toward Ca^2+^.

### FRET Determination in an Intracellular Environment

**Figure 5:**
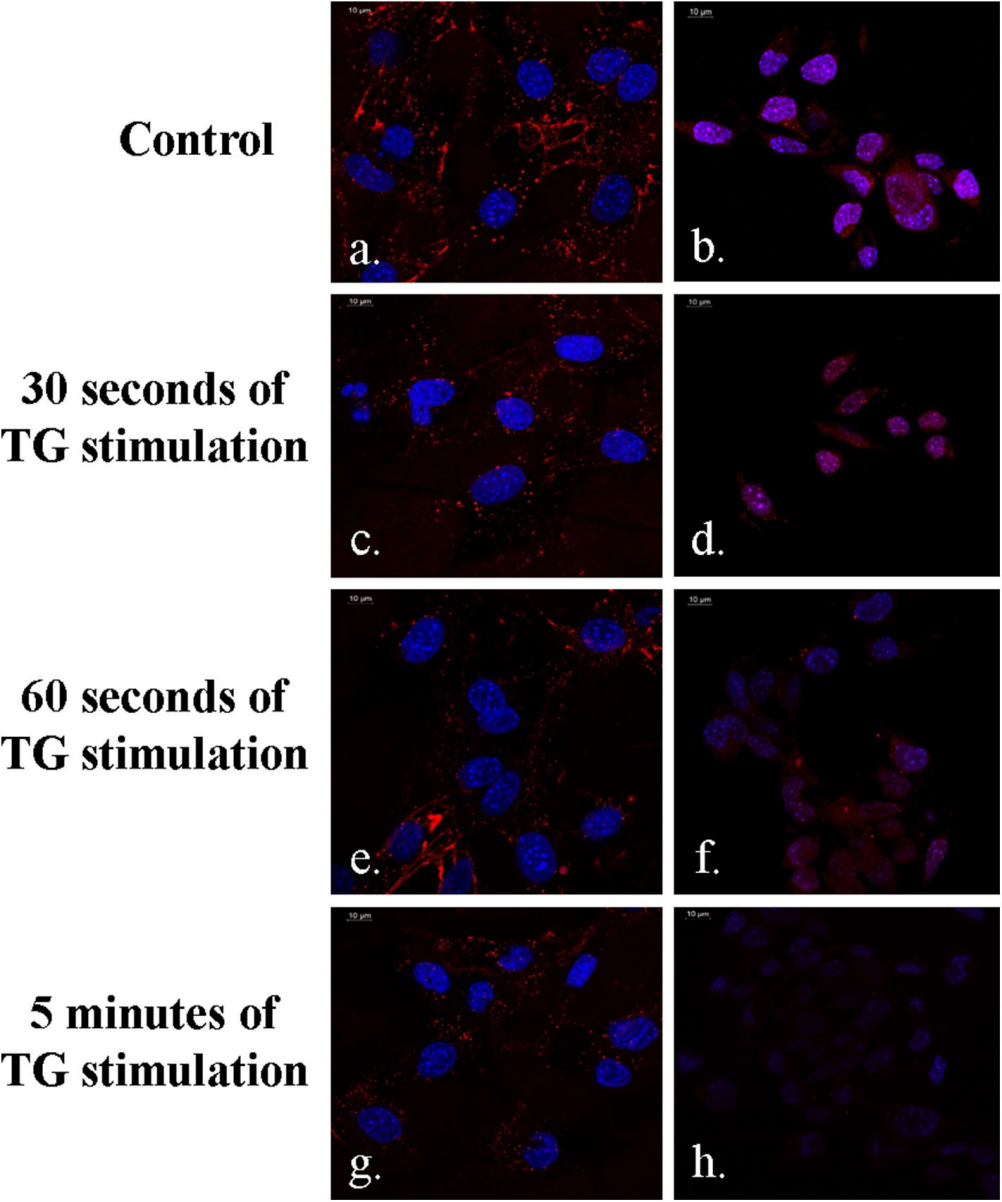
Demonstration of FRET in MC3T3 cells in the presence of the DSS conjugated aptasensors **(a–h)**. Fluorescence emission is minimum at 5 min of TG stimulation **(g,h)**.

The DSS peptide is a cell-penetrating peptide, which is composed of amino acids like aspartic acid and serine. It allows successful endocytosis of the DNA aptamer-based molecular beacons, and this has been shown in Figures 5a–h, which clearly indicates the red emission from the 655 nm QDs in the DSS-conjugated molecular beacons. A higher-intensity QD emission from the MC3T3 cells was observed in Figures 5a,b in the absence of TG stimulation. This sample has been indicated in the figure as control. TG stimulation increases intracellular calcium release, during which a greater number of aptasensors bind to the higher concentration of Ca^2+^, causing greater reduction in the fluorescence emission. As can be seen in Figures 5c–h, with an increase in the duration of TG stimulation, there was an elevation in the concentration of intracellular Ca^2+^. This decreased the fluorescence emission from the QDs with the progression of time and resulted in successful quenching in the presence of the target ion.

**Figure 6:**
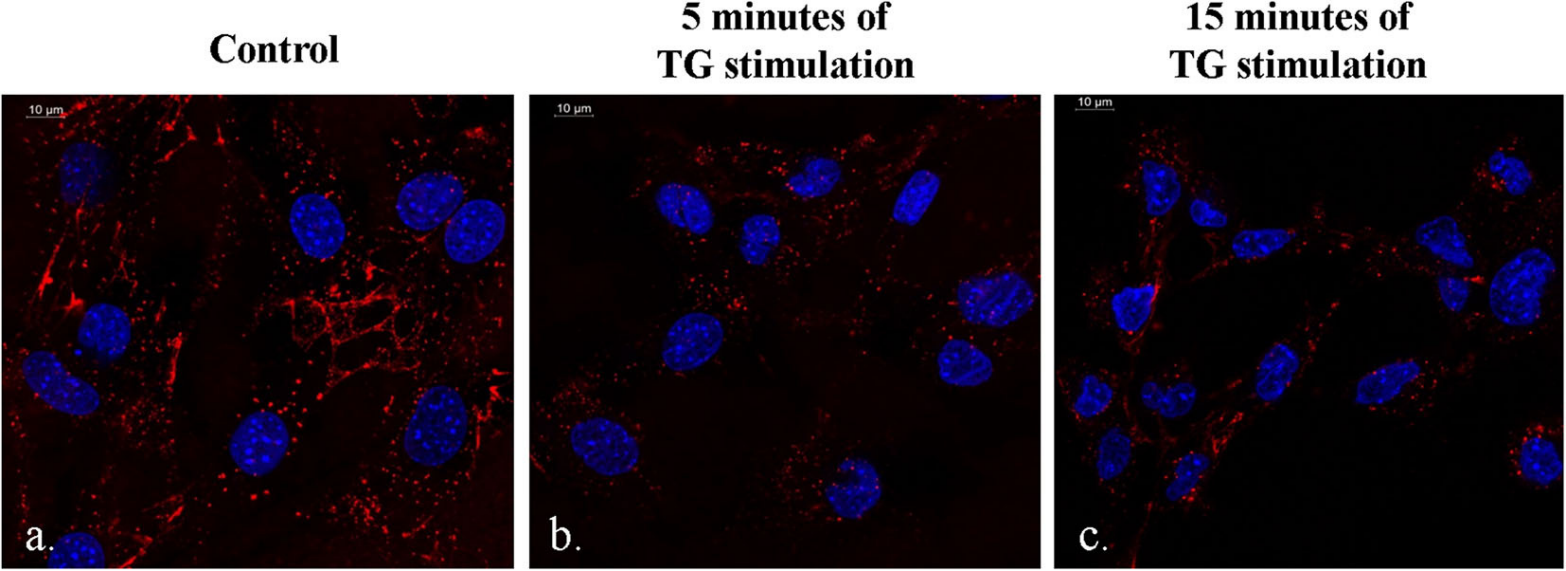
Difference in the quantum dot emission from the DSS-conjugated molecular beacon in the presence of no TG stimulation **(a)**, 5 min of TG stimulation **(b)**, and 15 minutes of TG stimulation **(c)**.

It was observed that there was a slight increase in the QD emission from the DSS-conjugated molecular beacon when the duration of TG stimulation reached 15 min (Figures 6a–c). This phenomenon could be attributed to the structure of the DNA aptamer, which reorganizes itself to a G-wire formation. During this process, there is a possibility that the donor–quencher pair is driven away, causing a slight decrease in quenching. However, the difference in QD emission is not very high between 5 and 15 min (Figures 6b,c). Additionally, the phenomenon of FRET is still evident between the control sample and the sample with 15 min of TG stimulation (Figures 6a,c).

## Conclusion

This study reports a simple DNA aptamer-based optical sensor, which detects Ca^2+^ between 0 pM and 35 nM. It employs a DNA aptamer as the primary Ca^2+^ sensing element and operates on the principle of FRET. It has the ability to detect the target ion up to a lower limit of 3.77 pM. Primary advantages of this sensor lie in its ability to successfully detect Ca^2+^ in an intracellular environment when used in conjunction with a cell-penetrating peptide (DSS). Additionally, it has very low sample requirement (5 μl) and is quite flexible, i.e., the same sensing strategy can be used to detect other analytes by replacing the sensing element with a DNA aptamer specific to the analyte.

## Data Availability Statement

The original contributions presented in the study are included in the article/supplementary materials, further inquiries can be directed to the corresponding author/s.

## Author Contributions

MS and MD conceived and advised on the overall project idea. AG conceived the cell culture experiments. SG and YC performed the experiments and wrote the manuscript. All authors read and edited the manuscript.

## Conflict of Interest

The authors declare that the research was conducted in the absence of any commercial or financial relationships that could be construed as a potential conflict of interest.
